# Early communicative gestures in human and chimpanzee 1-year-olds observed across diverse socioecological settings

**DOI:** 10.3758/s13420-022-00553-1

**Published:** 2022-11-28

**Authors:** Kim A. Bard, Takeshi Kishimoto

**Affiliations:** 1 Department of Psychology-King Henry Building, University of Portsmouth, King Henry I Street, Portsmouth, PO1 2DY UK; 2 University of the Sacred Heart, Shibuya, Tokyo, Japan

**Keywords:** Great apes, Infancy, Communication, Mutual gaze

## Abstract

We investigated the communicative gestures used by chimpanzee and human infants. In contrast to previous studies, we compared the species at the same age (12–14 months) and used multiple groups living in diverse socioecological settings for both species. We recorded gestures produced by infants and those produce by others and directed toward infants. We classified the gestures into the following types: human-usual, chimpanzee-usual, and species-common; and searched for within species and between species differences. We found no significant differences between groups or species in overall rates of infant-produced or infant-received gestures, suggesting that all of these infants produced and received gestures at similar levels. We did find significant differences, however, when we considered the three types of gesture. Chimpanzee infants produced significantly higher rates of chimpanzee-usual gestures, and human infants produced significantly higher rates of human-usual gestures, but there was no significant species difference in the species-common gestures. Reports of species differences in gesturing in young infants, therefore, could be influenced by investigators’ choice of gesture type. Interestingly, we found that 1-year-old infants produced the gesture of “hold mutual gaze" and that the chimpanzee infants had a significantly higher rate than the human infants. We did not find strong evidence that the specific types of gestural environment experienced by young infants influenced the types of gestures that infants produce. We suggest that at this point in development (before human infants use lots of speech), nonverbal communicative gestures may be equally important for human and chimpanzee infants.

## Prologue

The 1980s were an exciting time in developmental psychology, with discoveries of the preverbal communicative capacity in the human species. The 1980s were also an exciting time in comparative psychology, as we learned about the communicative capacity of great apes, through the numerous ape-language studies and the long-term field studies, especially by Goodall. I (K.A.B.) was fortunate indeed to begin my graduate studies at Georgia State University during this exciting time, while renting a room in Sally Boysen’s house. Sally regaled me with many stories of her experiences with chimpanzees, from their strength, their humor, their strategic social negotiations, and their capacity for strong social bonds (with humans as well as other chimpanzees). We discussed the different perspectives of ape-language studies, and what it meant to be communicatively competent.

After these conversations with Sally, while reflecting on what I learned about chimpanzees from her, I was reading Sacks (1985) book about humans’ need to be understood communicatively. I was working with the young chimpanzees in the Yerkes nursery, and watching how they interacted with other people, and these experiences led me to consider the importance of the co-construction of communicative meaning in chimpanzees. I recall observing nursery-reared chimpanzees of 3 and 4 years of age, very desirous of play, trying to get the human staff to play with them, especially chase play. The staff, however, had worked hard all morning, and by early afternoon were invested in resting. Initial attempts by the chimpanzees to engage the staff in play were relatively polite and resulted in some tickle play. But the chimpanzees wanted to be chased. The chimpanzees tried the relatively polite attempts—for example, taking an object from the caretakers and running away with it—but when this didn’t result in chase play, the young chimpanzees engaged in ever more intrusive and insistent behavior. They would climb on the fence above the staffs’ seated position, dangle their legs, and then drop down on their heads, quickly running away with a playface. Although the staff might simply yell, or shake a fist, the first or even the second or third time, once the chimpanzees had dropped on their head a sufficiently annoying number of times, the staff would jump up, and angrily run after the chimpanzees, much to delight of the chimpanzees—very happy to finally be engaged in chase play. At the time, when the initial behaviors did not serve the chimpanzees desires, my impression was that the chimpanzee acted like they thought that either the staff did not understand what they wanted or that they, themselves, had not asked in the right way. To the chimpanzees, it seemed perfectly sensible to keep trying to find a way to communicate their desires. I once tried to convey this to the staff: “You know that the chimpanzees jump on your head because they think that is the way to ask you to chase them,” but the staff did not seem to appreciate this remark or me going on to tell them that they could instead encourage more polite and agreeable communication. I was struck with the chimpanzees’ continuous striving to be understood via gestural communication, and their flexibility in co-constructing meaning with gesture.

Thus, I began my career with the knowledge that chimpanzees were sentient beings, with personalities and natural abilities to communicate nonverbally. This article is dedicated to Sally, with thanks for the enormous contribution she has made to my understanding and appreciation of chimpanzees.

Chimpanzees and humans share a long evolutionary history. It is therefore possible that these species may share commonalities in nonlinguistic communication, either in types of communication or in developmental processes from which communication emerges. In particular, the gestures of 1-year-old human infants may have similarities with those of 1-year-old chimpanzee infants. One aspect of importance in development may be the types of care experienced by infants, which can vary widely within species. For example, in the wild, chimpanzee infants spend much of their time in contact with their mothers (e.g., Goodall, 1986; Nishida, 1990, 2012), whereas in some captive settings, they may spend much of their time separated from their caregivers and placed on their backs, experiencing face-to-face contact with human caregivers from an early age (e.g., Bard, 1996). Similarly, human infants can experience much of their first year in physical contact with caregivers, including the mother (Crittenden & Marlowe, 2013; Meehan & Hawks, 2013: Keller, 2007), whereas in other settings, they can experience independence from physical contact with increased amounts of face-to-face contact (Bard et al., 2021; Takeshita et al., 2009). Early experiences of proximal caregiving (e.g., wild chimpanzees and foraging people) may engender more tactile communication in infants, whereas early experiences of distal caregiving (e.g., laboratory-reared chimpanzees and Western middle-class humans) may engender more visually based communication in infants. It would be interesting to examine whether these differences in early caregiving environments relate to differences in communication both within and between species, as this would provide insight into the impact of caregiving environments on the development of infant communication, illuminating similarities and differences in the early communication of these two closely related species.

There is a great deal of evidence that both human and chimpanzee infants communicative nonverbally, with gestures and sounds. There are a few studies that have shown cultural variation in the types of gestures used by human infants (e.g., Abels, 2020; Kwon et al., 2018), and in the frequency of some gestures in human infants (Blake et al., 2003; Salomo & Liszkowski, 2013). There are a few studies that have demonstrated that infant chimpanzees use communicative gestures in captive settings (e.g., Bard, Dunbar, et al., 2014b; Tomasello et al., 1994; Tomasello et al., 1985) and in the wild (Brundl et al., 2020; Plooij, 1979; van Lawick-Goodall, 1968), albeit most field studies of chimpanzee gestures do not focus on, or include infants (e.g., Hobaiter & Byrne, 2011; Roberts et al., 2012, respectively). There are a few studies that have documented cultural differences in gestures among wild chimpanzees, but these focus on adult gestures (e.g., grooming hand clasp: Van Leeuwen et al., 2017; Wrangham et al., 2016; McGrew & Tutin, 1978; directed scratching for grooming: Wilke et al., 2022; a variety of behaviors that serve to attract attention to the self, such as leaf-clipping: Bessa et al., 2022; Whiten et al., 2001; and making noise by leaf stripping or branch bending to threat or warn, respectively: Whiten et al., 2001). Few studies compare gestures in human infants with gestures in chimpanzee infants.

Matching the age in cross-species comparisons is important for a variety of reasons (Bard & Leavens, 2014). Studies of prelinguistic gestures in humans focus on infants, therefore comparative studies ought to focus on infants as well (i.e., matching the groups on at least one variable: Leavens et al., 2019). The most relevant reason, for the current study, is to match the groups in amount of early experiences and stage of life. There are few studies that have directly compared gesture use between young chimpanzees and young humans (e.g., Kersken et al., 2019). As far as we know, however, the current study is the first study of gesture use that directly compares multiple, diverse groups of human infants to multiple, diverse groups of chimpanzee infants. Using multiple groups is essential to capture the diversity within each species (e.g., Bard et al., 2021). Given that there is diversity within species (e.g., cultural differences or differences associated with early rearing: reviewed in Bard et al., 2021) then comparing one group of humans (that may not be representative of the entire human species: e.g., Kline et al., 2018) to one group of chimpanzees (that may not be representative of the entire chimpanzee species; Bard & Leavens, 2014; Leavens et al., 2019) is fraught with confounds and is a design that does not isolate species membership as a causal variable. Thus, designs that embrace diversity for both species are more helpful than 1 group–1 species designs to indicate species differences.

## Within-species studies of human infant gestures

Among human infants, we know that there are cultural differences in the frequency or the prevalence of gestures, such as, point, show, offer, wave, and demonstrate. We also know that the vast majority of studies of infants, regardless of the topic area, have been conducted in *W*estern, *E*ducated, *I*ndustrialized, *R*ich and *D*emocratic (i.e., WEIRD: Henrich et al., 2010) settings (Nielson et al., 2017). Fewer rural Mayan infants pointed compared with urban Dutch and urban Chinese infants (Salomo & Liszkowski, 2013), although other studies found no cultural differences in pointing (e.g., among infants from Canadian, rural Peru, and rural India settings: Callaghan et al., 2011). The frequency of requesting gestures varies with socioeconomic status (SES), with more requesting in infants from high SES families compared with low and medium SES families (Abels & Hutman, 2015), and there may be changes in frequency with age. In a study in Japan, for example, requesting gestures decreased from 9 and 14 months of age (Blake et al., 2003). It seems that “show” is particularly evident in urban dwelling infants (perhaps associated with distal caregiving), since no show gestures were observed in rural Nigerian infants (Childers et al., 2007). “Show” can be when an infant holds an object for the partner to look at (which can be labelled as “show object”) or when highlighting an unusual aspect of the self, as in mirror-self recognition studies (which can be labelled as “show self”). A significant difference was found between the frequency of toddlers showing the face (the marked spot in mirror-recognition studies) between urban (German and India) compared with rural (Indian and Nso) communities (Kärtner et al., 2012). In naturalistic observations, there is a relatively low frequency of showing objects by infants, albeit at significantly higher rates in urban (Chinese and Dutch) than in rural (Mayan) settings (average of eight, five vs. two per hour, respectively: Salomo & Liskowski, 2013). Notably low levels of showing were observed in free play between infants and mothers in an urban Japanese community (Blake et al., 2003).

Offering may be considered a gesture focusing on social etiquette, with strong cultural differences (Lancy, 2015). In many Western middle-class families, offering is part of a give-and-take exchange, perhaps with a goal of sharing attention on objects (e.g., Tomasello, 2019), whereas in many other communities, offering is part of the sharing etiquette of giving to others (e.g., Bakeman et al., 1990; Lancy, 2015). Offering an object, thus, may mean, “Let’s exchange the object between us” in the first instance, or maybe mean, “Here is an object for you to have” in the second instance. Although in both instances an object is offered, in the first the infant expects the object to be returned, but not in the second. This difference in meaning in the give/offer gesture may explain why Salomo and Liskowski (2013), for example, did not find cultural group differences in the frequency of the offer gesture.

Another gesture with potentially different meanings is “arm raise.” In many WEIRD settings, this gesture is interpreted to mean that the infants want to be picked up (this was used as the prototypical example of the process of ontogenetic ritualization by Tomasello & Call, 2007; see Bard, Dunbar, et al., 2014b, for further details). This gesture may appear less often in cultural communities in which adults anticipate infants’ needs (i.e., in which infants are not expected to signal their needs: Bard et al., 2021; Morelli et al., 2017) or in cultures in which caregivers sit together with infants rather than bringing infants up to the level of standing caregivers (Kwon et al., 2018). In the comparative literature, the arm-raised gesture is often interpreted as an invitation for play, for chimpanzee infants living in captive settings and wild settings (e.g., Bard, Dunbar, et al., 2014b; Graham et al., 2018; Plooij, 1979; Tomasello et al., 1994; van Lawick-Goodall, 1968), but can be interpreted as a threat (Goodall, 1986), or as an invitation for grooming (Plooij, 1979). Differences in meaning of the arm raised gesture can be explained, in part, with knowledge of the movement trajectory of the arm (the raised arm moving down is a play invitation, whereas the arm being raised up is a threat, and the arms held in a static vertical position indicate the pick-me-up gesture) and positioning of the arm/hand (e.g., open hand for play invitation, back of the wrist for threat: Bard, 1996; Goodall, 1986). Rather than both arms, more often it is a single arm that is raised as a request for grooming (and soliciting grooming with a clasping hands gesture, which occurs only in some chimpanzee communities, is also single arms raised [McGrew & Tutin, 1978], but is not a skill observed in infants [Nakamura & Nishida, 2013; Wrangham et al., 2016]).

There are additional gestures that focus on social etiquette, which may be very prevalent in some, but not all human cultures. For example, the finger to the lips with the “shh” sound (labelled the Quiet gesture by Kwon et al., 2018) is a gesture with the conventionalized meaning for others to be quiet. Wagging the finger or shaking the head may both mean “no, no,” but the former usually is an admonishment to others, whereas the latter is usually an individual’s refusal (reject or protest: Blake et al., 2003). These and other gestures of social convention are not often included in human infant gesture studies, perhaps because it does not involve an object, and thus may be more difficult to be classified as a referential or intentional gesture. Similarly, some chimpanzee studies include gestures involving social manners such as rump or wrist presents (sometimes called wrist offer: Tomasello et al., 1994), which can be interpreted as acknowledgements of rank relationships or appeasements (e.g., Bard, Dunbar, et al., 2014b). Perhaps because the outcome of this gesture may be the lack of aggression; for example, rather than an observable outcome, this gesture may be missed in some studies of chimpanzee gestures.

## Within-species studies of chimpanzee gestures

A number of studies have documented the repertoire of gestures used by chimpanzees (Hobaiter & Byrne, 2011, 2014; Liebal et al., 2004; Roberts et al., 2012; Tomasello et al., 1985; van Lawick-Goodall, 1968) and bonobos (Graham et al., 2018; Pika et al., 2005). It has been shown that chimpanzees and bonobos share about 90% of their gestures (Graham et al., 2018) and that young chimpanzees emit many gestures in the contexts of play (Fröhlich et al., 2016a), food begging (Fröhlich et al., 2020; van Lawick-Goodall, 1968), grooming (Bard, Dunbar, et al., 2014b), and travelling (Fröhlich et al., 2016b).

A few studies have documented within-species differences—for example, in gesture rates (higher rates in infants living in Kanyawara [Kibale National Park, Uganda] compared with Tai [Ivory Coast]: Fröhlich et al., 2017), in repertoire size (significantly related to number of interaction partners: Fröhlich et al., 2017), and in the presence or absence of specific types of gestures, some indicating cultural customs (e.g., Whiten et al., 1999) and others indicating flexibility (Fröhlich et al., 2016a, 2016b). Another dimension of within-species differences is in the meaning of a gesture. For example, exaggerated loud scratches (Wilkes et al., 2022) appear to have different meanings across wild chimpanzee sites: including “Groom me here” (in the Ngogo fieldsite: Pika & Mitani, 2006), “Please groom me” (in two field sites: Sonso, Kanyawara), and “Let me groom you” (in the Kanyawara field site). We agree with the conclusion of Fröhlich et al. (2017): “Hence, communicative abilities rely on a combination of social, physical, and cognitive development in the individual while interacting with the social and physical world surrounding it” (p. 278).

## Cross-species studies of infant gestures

There are just a few studies that have directly compared the gestural repertoires of human infants/juveniles to chimpanzee infants/juveniles. For example, Kersken et al. (2019) found that 54% (28 of 52) of the gestures found in wild chimpanzees, aged 0–5 yrs, from Budongo, Uganda, were shared by human 1–2-year-olds (urban German combined with rural Ugandan infants). The most frequently observed gestures produced by human infants were “directed reach” (i.e., point), “reach palm” (i.e., request), “arm raise,” and “throw object,” which are also reported in chimpanzees living in both captive (e.g., Primate Foundation of Arizona: Berdecio & Nash, 1981; Yerkes Primate Center: Leavens, 2021; Tomasello et al., 1994) and wild settings (e.g., Budongo: Byrne et al., 2017; Gombe: van Lawick-Goodall, 1968; Tai & Kibale: Fröhlich et al., 2016a, 2016b). Additionally, communicative milestones of both wild (Brundl et al., 2020) and captive chimpanzees (Bard, 2017) appear very similar to those of humans in the first 5 years of life.

Although previous comparative studies have sometimes made comparisons among great ape groups (e.g., Kersken et al., 2019), no previous comparative study of gestures has investigated within-species variation in both human and chimpanzee infants. Additionally, we are not aware of any comparative study that has investigated both human-typical gestures and chimpanzee-typical gestures when comparing human with chimpanzee infant samples. Here we include both those gestures typically studied in human infants (sometimes called conventional gestures, such as pointing), and those typically observed in chimpanzee infants. Moreover, we compare three groups of chimpanzee infants living in diverse settings and three groups of human infants living in diverse settings, to investigate both within species and between species variation. We hasten to add, however, that our sample sizes were small. Therefore, this study can be interpreted as supporting (or rejecting) the premise that there are within-species variations in gestures, since we studied infants of each species that lived in diverse settings, but further studies will be required to support the generalizability of our findings.

## Classification of gestures

There are many ways to classify gesture types. In chimpanzee studies, classifications range from classifying structural elements (e.g., Hobaiter & Byrne, 2011; Plooij, 1984; Roberts et al., 2012), or classifying gestures in terms of the context in which they occur, to classifying meanings (Bard, Dunbar, et al., 2014b; Goodall, 1986). For example, suppose an individual X approaches another (Individual A) who is of higher rank/dominance (context). At the level of structure elements, Individual X may display a “fear grin,” may present their rear end (or wrist), and/or may emit a specific type of pant-grunt vocalization directed at Individual A. One could simply classify these as “submissive” gestures, since they were displayed in this context. If attending to the meaning, one could classify the different elements (i.e., that there was an indication of emotional arousal [fear grin] or that there was a gesture indicative of social etiquette [rump or wrist present] and/or there was a gesture indicative of dominance relationship [vocal pant-grunt] when given by Individual X indicates an acknowledgement that Individual A is dominant). Meaningfulness of gestures is sometimes assessed by determining the ASO (apparently satisfactory outcome: Hobaiter & Byrne, 2014), or the communicative message (e.g., Bard, Dunbar, et al., 2014b). We felt that it was important to distinguish gestures according to meaning, at the moment of occurrence, in part, because one cannot know what is being communicated after the fact, especially if only structural elements are record (e.g., touches to specific body locations or even specific touch-location configurations do not convey specific meanings: Bard et al., 2019). We therefore choose to record the functional meaning of each gesture when it was observed. We recorded only those behaviors with communicative meaning or communicative intent but did not limit this to manual gestures.

We note that the same gesture may have different meanings across contexts and across cultural settings. For example, pointing in WEIRD settings may appear as part of a “natural pedagogy” (Csibra & Gergely, 2011), interpreted to mean that the infant is inviting instruction (e.g., pointing to a picture in a book invites the parent to name the object: Liszkowski et al., 2004). Pointing is found commonly among captive chimpanzees, with most studies finding more than half the subjects produce points, comprehend points, and are sensitive to partner’s attention during pointing events (Krause et al., 2018). Pointing is rare but present in wild chimpanzees as well (e.g., Budongo: Hobaiter et al., 2014). Leavens (2021) suggests that pointing develops in both species when individuals encounter the referential problem space. The problem space is when an individual desires an object but there is a barrier to obtaining it directly. It becomes a referential problem space, when the individual has the capacity to refer both to the desired, but out-of-reach object and to a communicative partner willing to fulfil the request (Leavens et al., 2005). This is the only current theory that explains both the cross-species and within species differences in propensity to point (Leavens, 2021). Lancy (2015) argues from the historical cross-cultural database that pointing in human infants is rare and caregivers may not respond to it. Much is made of ostensive cues, especially eye gaze, signaling the intentionality of infants’ communicative gestures (e.g., using a WEIRD framework: Gomez, 1996), but there are human communities in which gaze is only directed down the social hierarchy and other human communities in which infants are attentive to multiple events at the same time, albeit not necessarily through gaze (reviewed in Bard et al., 2021).

The gesture of “show” can have many different meanings. Most common in the developmental literature is showing an object. However, also common in infants living in middle-class, urban settings is “showing self,” which, similar to showing an object, is highlighting something as special. Showing self is defined as infant highlighting some (unique) feature on the infant’s own body (e.g., when toddlers have mark on their face: Kärtner et al., 2012), or in the infant’s own actions. It is interesting that in the anthropological literature, “show” does not refer to an infant gesture but rather to caregivers parading through the village while holding the infant facing outward to an admiring crowd (Lancy, 2015) or making infants look beautiful for others to behold—for example, to encourage child care by others in Beng communities (Gottlieb, 1995). In other human communities, infants are socialized to blend in, to be in harmony with the social group, and “showing off” or any behavior designed to make an individual stand out is rarely seen (Keller, 2007; Keller, 2018; Morelli et al., 2018; Yovsi et al., 2009).

## Current study

The current paper is neither a complete accounting of gestural repertoire types nor a complete description of repertoire size. Rather, it is a descriptive account of the production of communicative gestures based on approximately 45 minutes of natural observation for each of 51 infants collected originally for a study of joint attention (Bard et al., 2021). This study differs from many studies of human gestures but is similar to many studies of chimpanzee gestures in that it is based exclusively on observation. We think it is important to document gesture production during infants’ everyday interactions with others, in their everyday ecology, in order to understand the ecological situatedness of gestural communication (Dahl, 2017). We discuss the groups as embedded within their sociocultural ecologies (Keller & Kärtner, 2013; Rogoff et al., 2018), with the presumption that the behaviors of infant and caregiver(s) are adaptive to specific socioecologies (Fouts et al., 2016; Keller, 2018).

Our main aim is to document the extent to which we see variety in the types of gestures produced (and received) by infants across six samples, which vary in both socioecology and species membership. One hypothesis is that there will be species differences. Perhaps human infants will differ from chimpanzee infants in the frequency and range of gestural repertoire due to the higher levels of communicative competence of the human species. Alternatively, chimpanzee infants, without spoken language, may rely more on nonverbal gestural communication than do human infants. Our hypothesis, however, is that there will be diversity in the gestures within each species, given that we are sampling quite diverse groups (see Bard et al., 2021, for further details).

## Methods

### Subjects and settings

#### Human infants

We used notes taken during the coding of triadic connectedness, as reported in Bard et al. (2021). To summarize, notes about gestures were obtained from 30 human infants, eight from urban settings near Universities in the south of England (UK sample, see Ross et al., 2007), 10 from hunter–gatherer communities of the Central African Republic (Aka sample, see Hewlett, 1991; Hewlett & Roulette, 2016), and 12 from communities of subsistence farmers in rural villages of North West Cameroon (Nso sample, see Keller, 2007; Nsamenang, 1992/2017; Otto & Keller, 2015). We chose these three settings primarily because they represent a wide diversity of socioecological settings in which extant humans live, and secondarily because we could obtain videotaped recordings of the everyday behavior of infants.

##### Socioecology of the human infants

The UK infants were videotaped mostly in their living rooms, most often with their mother, although all lived in two-parent families, some also interacted with an older sibling. Parents practiced distal caregiving. All UK families had many toys and books designed for infant play. Some had a grandmother in the household, but she was not present in the videotapes.

The Aka infants were videotaped outdoors, in the clearings outside of their mobile, beehive shaped huts. They had a network of caregivers (e.g., Meehan & Hawkes, 2013) and lived in a community of 25–35 individuals of mixed ages, mixed kin, and both sexes. Aka infants experienced proximal caregiving from many caregivers (Meehan & Hawkes, 2013), and ethnotheories of “fierce egalitarianism” (Gray, 2009). The Aka infants had access to rain-forest vegetation and to various cooking utensils and tools, but there were no dedicated infant toys.

The Nso infants were videotaped mostly in the communal clearings outside of their permanent mud brick houses. There was at least one social partner with the infant (albeit not always the mother), but usually there were between six and 45 people of mixed ages, and both sexes in the community with whom infants could interact. Nso infants experienced proximal caregiving from many caregivers (Keller et al., 2009), with ethnotheories of “hierarchical interconnectedness” (households were patrilineal: Keller, 2021). There were household objects, natural vegetation, and some animals available for infants to play with. Rarely a family had a toy, such as a ball.

#### Chimpanzee infants

We used notes taken during the coding of triadic connectedness, reported in Bard et al. (2021). To summarize, notes about gestures were obtained from 21 chimpanzee infants—12 chimpanzee infants from Gombe National Park, Tanzania; four chimpanzee infants were from Chester Zoo (Zoo: Ross et al., 2014); three infants from the Primate Research Institute, Kyoto University, Japan (PRI: Matsuzawa, 2003; Ross et al., 2014); and two chimpanzee infants were raised by an adult human female, mostly in a human home, with daytimes spent at the Chimpanzee Cognition Laboratory, The Ohio State University, Columbus Ohio, where there were other adult and subadult chimpanzees. The PRI and Zoo chimpanzees were combined due to the small samples and the lack of obvious group differences in outcomes. We chose these settings primarily because they represent a wide diversity of socioecological settings in which extant chimpanzees live, and secondarily because we could obtain videotaped recordings of the everyday behavior of infants living in these settings.

##### Socioecology of chimpanzee settings

The Gombe infants lived in the tropical rainforest of the Gombe National Park. The available social partners always included the biological mother, and other partners, including siblings, unrelated families, and adult males were often available. Gombe infants experienced proximal caregiving, within a fission-fusion community with a male dominance hierarchy (Goodall, 1986). There was natural vegetation available but no human artifacts for Gombe infants.

The Chester infants lived in a captive environment typical of most zoos, with an outdoor (ground) area and indoor (concrete floor) area containing climbing structures, canvas hammock-like areas, and hanging ropes. Chester infants experienced proximal caregiving and lived in a stable social group of 26 other chimpanzees, including peers, juveniles, adolescents, and adults of both sexes. The infants played on the hanging ropes and had a few plastic toys available for enrichment. Most of the videotaping took place while the infants were inside.

The PRI infants lived in a very enriched physical setting with an outdoor area containing a 30-m high climbing structure with ropes, tubes, and wooden platforms at different levels of the enclosure, and large indoor areas for sleeping. The infants experienced proximal caregiving from their mothers, lived in a stable group of 11 other chimpanzees (but without siblings or juveniles), and had distal interactions with an adult male human experimenter while participating in cognitive studies each weekday (e.g., Matsuzawa, 2007). Infants played with natural vegetation and ropes while outside, and some manmade objects and toys while inside. All of the videotaping of the PRI infants took place while they were outside.

The Home-raised infants lived in a Western middle-class home (overnight and weekends), and spent their days at the Chimpanzee Cognition Lab, The Ohio State University. These infants experienced distal caregiving, although the two infants were always together. At both locations there were lots of toys available for the infants. They were filmed in both the indoor rooms of the lab and on the grass outside the building.

### Videotaping procedure

Naturalistic observations were recorded of each infant while they interacted with their everyday ecology. There were no objects or specific instructions provided by the researchers. All infants were able to interact with their usual social partners in their usual manner, there was no structure imposed by the researchers. All infants were approximately 12- to 14-months of age when videotaped, but videotapes were made on different dates (see Table 4: Bard et al., 2021, p. 102, for an overview).

### Coding

While the main goal of coding these videotapes was to collect data on joint engagements (Bard et al., 2021), handwritten notes were made of any gestures that occurred, whether by infant or by others directed to infants, with appropriate contextual information. These gestures were then classified into the categories listed in Table 1. Reliability was assessed by two coders independently classifying a total of 156 gestures from the written notes of 15 human infants (UK *n* = 4, Aka *n* = 4, Nso *n* = 7) and 10 chimpanzee infants (PRI/Zoo *n* = 5, Gombe *n* = 3, Home-raised *n* = 2). The subjects selected for reliability (approximately half the total corpus) were randomly selected from the total corpus of 30 human infants and 21 chimpanzee infants. Cohen’s Kappa was 0.82, indicating excellent agreement (Bakeman & Quera, 2011).

**Table 1:** The list of infant gestures used in the current study, organized by type, with definitions and indicative citations

Type Gesture	Definition	Indicative citations
Human-usual		
Point	Extended arm with directionality indicated by either index finger or whole hand, typically held vertically.	Callaghan et al., 2011; Leavens, 2021; Salomo & Liszkowski, 2013; Wilkins, 2003
Show object	An object is held and moved into the visual path of the interactant, often with eye contact or gaze alternation	Choi et al., 2021
Offer object	An object is held in an open hand moved toward the partner, with the expectation that the partner will take it	Bakeman et al., 1990
Place object	An object is put down with a flourish to indicate that it is now ‘on display’	Salomo & Liszkowski, 2013
Wave	The hand is moved in a distinctive way to indicate Hello or Goodbye.	Kersken et al., 2019
Demonstrate	A specific action is directed to a particular object or activity with the expectation that the action will be copied by the partner	Abels, 2020; Hewlett & Roulette, 2016
Chimpanzee-usual		
Touch	A gesture that is tactile contact alone	Bard, Dunbar, et al., 2014b; Bard et al., 2019; van Lawick-Goodall, 1968
Food beg	Typically, a hand held palm-up near a social partner that is eating or holding food	Bard, Dunbar, et al., 2014b; van Lawick-Goodall, 1968
Request tickle	Hands over head (to tickle neck), or touching a particular part of the body (e.g., tickle under arm) or reaching to partner while displaying a playface	Bard, Dunbar, et al., 2014b; Plooij, 1979; van Lawick-Goodall, 1968
Request chase	Typically, a playful approach, followed by a quick run away with a look over the shoulder to partner. Grabbing and running away with an object can also be a gesture to indicate chase play	Bard, Dunbar, et al., 2014b; Tomasello et al., 1985; van Lawick-Goodall, 1968
Request groom	A number of behaviors could be used to request grooming from a partner, including one arm raised, with or without directed scratch, a particular posture, and sometimes accompanied with facial or vocal indices of grooming	Bard, Dunbar, et al., 2014b; van Lawick-Goodall, 1968
Appease	Any submissive behavior that is used in the context of saying sorry, reducing the perception of an affront, or attempting to calm conflict	Bard, Dunbar, et al., 2014b; van Lawick-Goodall, 1968
Rank-related	Unidirectional behaviors (up the hierarchy), including wrist or rump present, pant grunt (greeting vocalization)	Bard, Dunbar, et al., 2014b; Brundl et al., 2020; Laporte & Zuberbuhler, 2011; van Lawick-Goodall, 1968
Threat bark	A type of vocalization, sounding much like a sharp single cough, that can mean ‘Stop that!’ or ‘Go away!’	Bard, 1996; Bard, Dunbar, et al., 2014b; van Lawick-Goodall, 1968
Nurse	Any behavior directed to an adult female that indicates the infant desires to suckle. It can be a pat on the chest, a tug on clothes near the breast, or a head bop on the chest	Bard, 1996; Bard et al., 1990; Tomasello et al., 1985
Species-Common		
Arm raise	One or both arms are held up vertically for some seconds	Bard, Dunbar, et al., 2014b; Kersken et al., 2019; Plooij, 1979; Tomasello et al., 1985;
Travel	Behaviors involved in coordinating travelling, including travelling together, or soliciting help in travelling	Falk, 2004; Fröhlich et al., 2016a, 2016b; van Lawick-Goodall, 1968
Request comfort	Behaviors directed to a social partner with the goal of calming the self (e.g., asking for a hug), directed whimper	Bard, Dunbar, et al., 2014b; van Lawick-Goodall, 1968
Grunt	Nonverbal vocalization that is not linked to distress or rank or appeasement, but directed to a social partner	Kersken et al., 2017; McCune et al., 2021
Whine	Any distress vocalization, including crying, whimpering, and fussing.	Bard, 1996
Request help	Any sort of behavior that functions to aid the signaller, including accessing objects, aiding in travel, negotiating social relations	Bard, Dunbar, et al., 2014b
Other	Any other gesture, including pretend play, imitation, showing off self, and refusals/protests	Bard et al., 2021; Blake et al., 2003; Hayes, 1951; Kärtner et al., 2012
Hold mutual gaze (Hold MG)	Infant and a social partner hold eye contact for 2 seconds or more	Blake et al., 2003; Byrne et al., 2017; Csibra & Gergely, 2011; Gomez. 1996; Kersken et al., 2019

The references are not exhaustive but are indicative with priority given to those that are culturally inclusive

### Data analytic strategy

There were individual differences in the amount of time infants were visible in the videotaped observations. For example, the Gombe chimpanzee infants lived in the tropical rain forest, where visibility was sometimes very limited (Goodall, 1986). Infants were very small, making them harder to see than adults. At Gombe, although a family might have been the focus for many hours of videotaping, the infant was the main focus for only part of the session. For these reasons, we analyzed the rates of gesturing (number per 10 minutes of visible time) to compare groups given the relatively infrequent nature of infant gesturing.

Initially, we analyzed the extent to which there were differences among the six groups in overall rates, and if the omnibus test was significant, specifying with contrasts which groups differed significantly from the others. If there were no significant differences across the groups, we investigated whether there were significant differences between species (i.e., all the human infants compared with all the chimpanzee infants). We analyzed infant-produced gestures (the main focus of the study) and also infant-received gestures, when mothers and/or others directed a gesture to the focal infant. We also assessed whether there was any relation between them with correlations to determine if rates of infant-received were related to infant-produced gesture types.

After data were collected, we analyzed one gesture (hold mutual gaze) on its own because (1) we were surprised to find that it occurred in our sample of 1-year-olds, and (2) we thought that this gesture, in particular, might differ in infants who had experienced proximal caregiving versus those that experienced distal caregiving (Bard et al., 2021; Keller, 2007). In other words, given that this behavior occurred, we wanted to know if there were within species differences, and whether this gesture was related to any particular type of infant-produced or infant-received gesture.

## Results

### Total gestures (frequency / 10 minute)

**Fig. 1:**
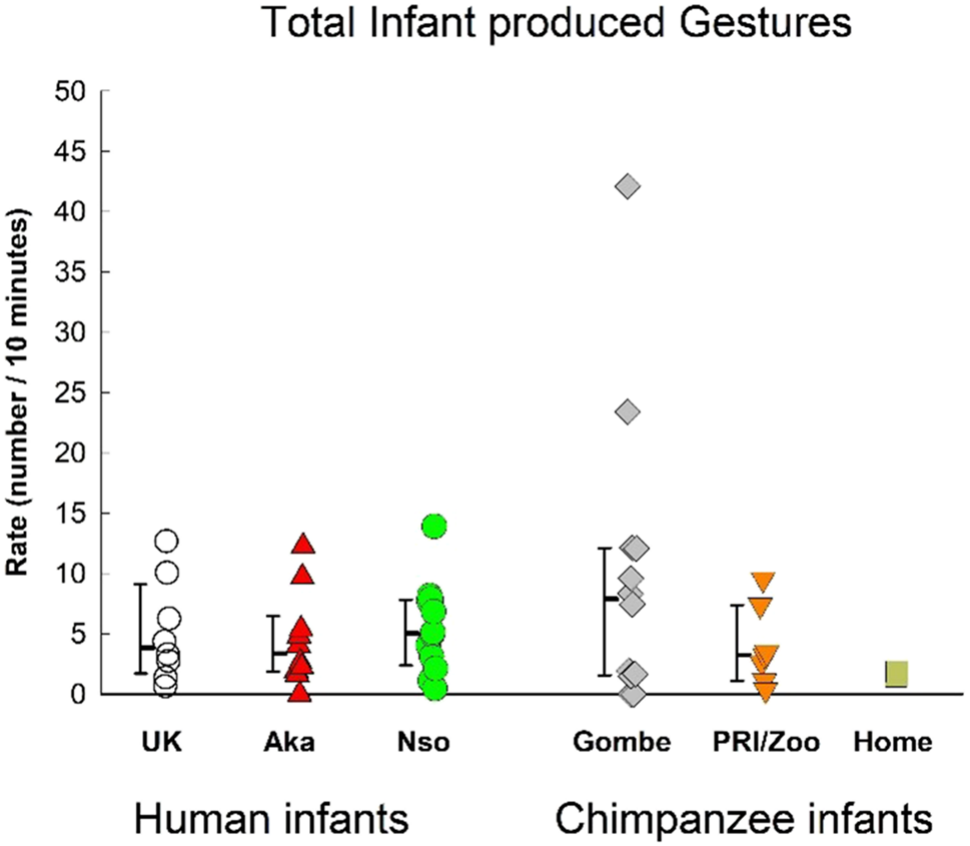
Infant-produced gesturing in the three human samples (UK, Nso, and Aka) and the three chimpanzee samples (Gombe, PRI/Zoo, and Home-raised). Individual rates (number per 10 minutes of visible time) are shown with medians and 25–75% confidence intervals for each sample

**Fig. 2:**
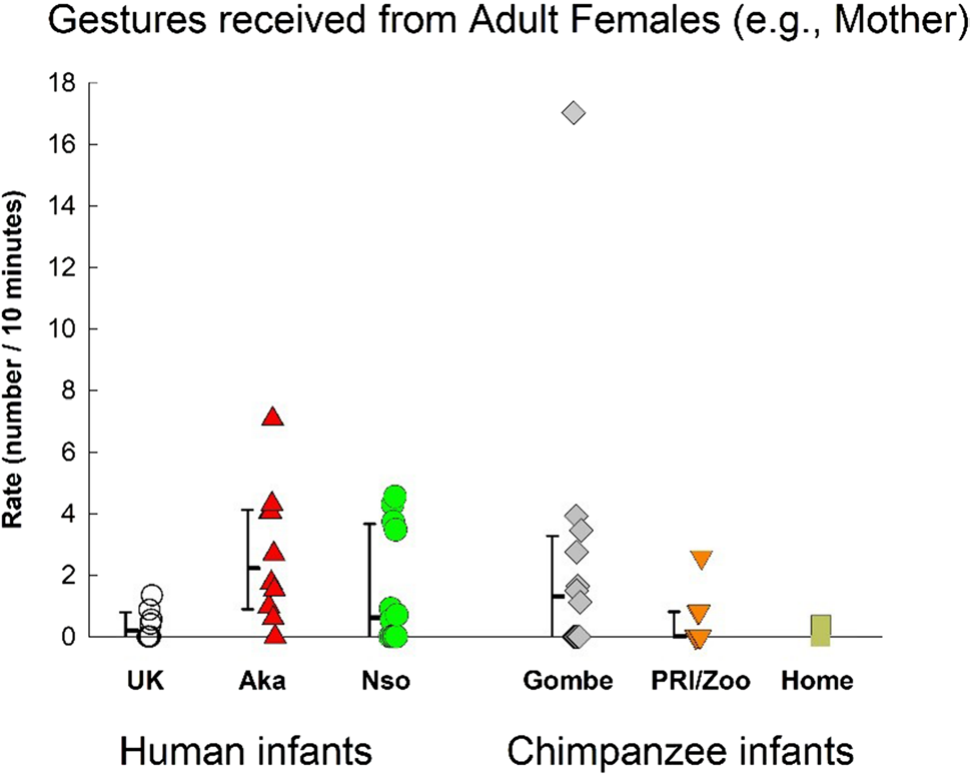
Total gesturing received by the focal infants from adult females in the three human groups (UK, Nso, and Aka) and the three chimpanzee samples (Gombe, PRI/Zoo, and Home-raised). Individual rates (number per 10 minutes of visible time) are shown with medians and 25–75% confidence intervals for each sample

**Fig. 3:**
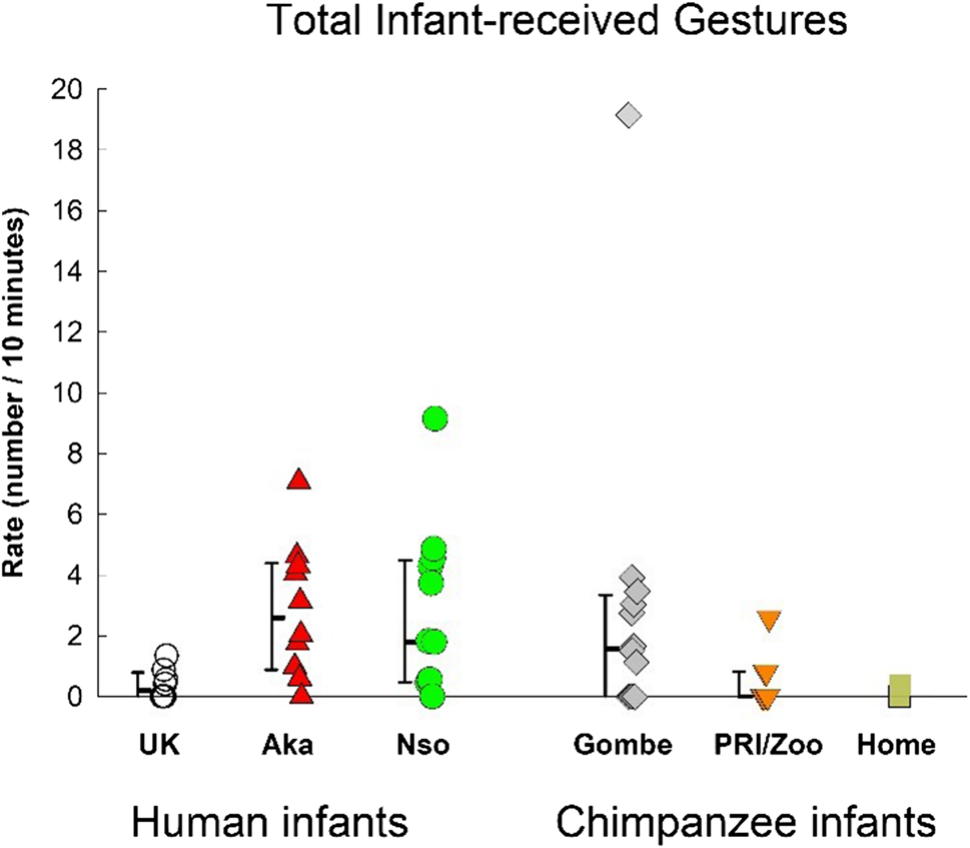
Total gesturing from mothers and others (infant-received gestures) in the three human samples (UK, Nso, and Aka) and the three chimpanzee samples (Gombe, PRI/Zoo, and Home-raised). Individual rates (number per 10 minutes of visible time) are shown with medians and 25–75% confidence intervals for each sample

There was not a significant difference among the samples in gesture rates, as determined with the omnibus multivariate ANOVA (i.e., with overall infant-produced rate and overall infant-received rate), *F*(10, 90) = 0.253 *p* =.239, η_p_^2^ =.127 (Figs. 1, 2 and 3). Additionally, there was not a significant species difference, *F*(2, 48) = 0.32, *p* = .455, η_p_^2^ = .032. This means that the overall rate of infant-produced gesturing was similar across all groups, and across both species, and that the overall rate of infant-received gesturing was also similar across all groups and across both species.

### Different types of infant-produced gestures

**Fig. 4:**
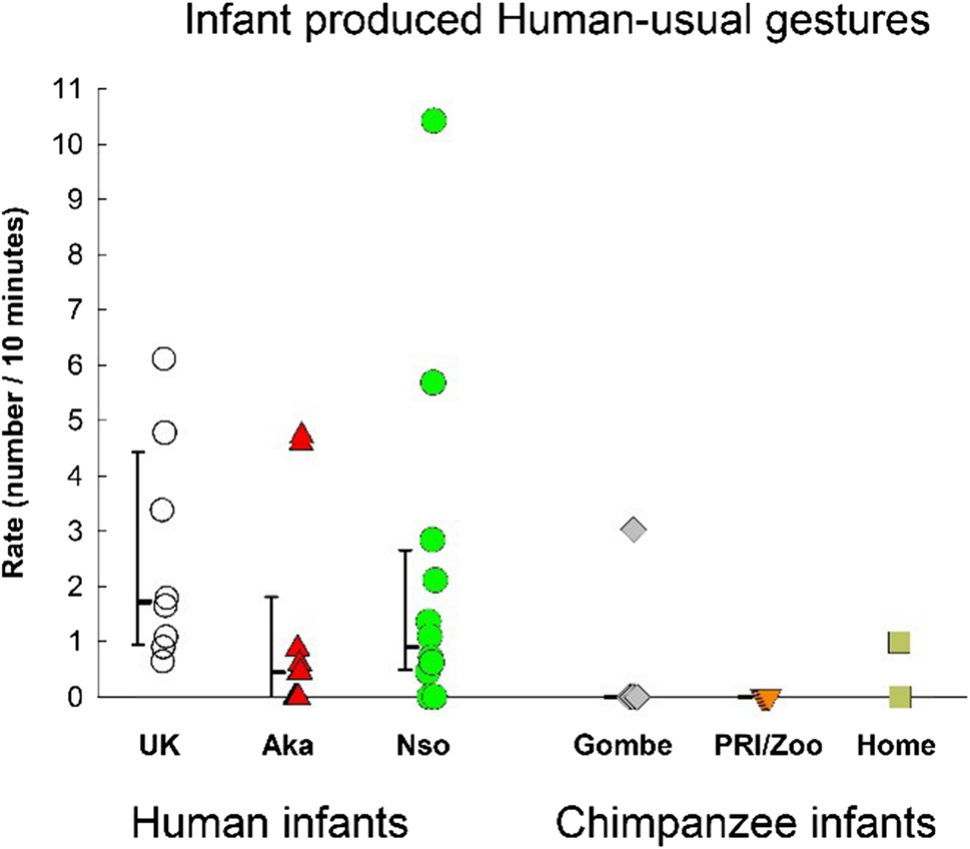
Human-usual gestures (i.e., those usually reported for human infants) produced by infants in each of our three human samples (UK, Nso, Aka) and our three chimpanzee samples (Gombe, PRI/Zoo, Home-raised). Individual rates (number per 10 minutes of visible time) are shown with medians and 25–75% confidence intervals for each sample

We considered gestures that are usually reported in the developmental literature as “human-usual” gestures (i.e., point, show, place, offer, wave, demonstrate). “Place,” however, did not occur in our observations and was dropped from the computation. The rates of human-usual gestures in each group are shown in Fig. 4. There was a significant group difference, *F*(5, 45) = 2.501, *p* = .044, η_p_^2^ = .217. The rate of infant human-usual gesturing was highest in the UK group. The rate in the UK sample was significantly higher than the rate in the Gombe sample, *p* = .013, and the PRI/Zoo sample, *p* = .015. but there was no significant difference between UK and Nso, UK and Aka, UK and Home-raised chimpanzees.

**Fig. 5:**
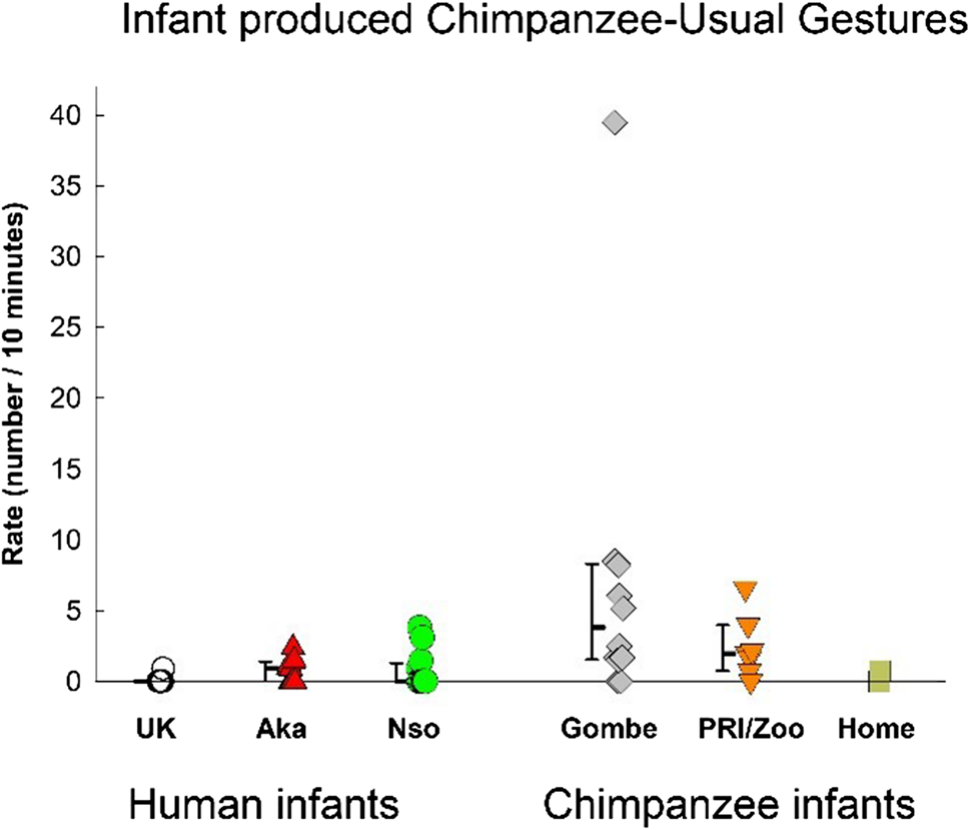
Infant-produced chimpanzee-usual gestures (those usually reported for chimpanzees) in each of three human samples (UK, Nso, Aka) and the three chimpanzee samples (Gombe, PRI/Zoo, and Home-raised). Individual rates (number per 10 minutes of visible time) are shown with medians and 25–75% confidence intervals for each sample

We considered “chimpanzee-usual” gestures as those that are usually reported in the comparative literature (i.e., Food Beg, Request groom, Request tickle play, Request chase play, Rank-related, Appease, ThreatBark, and Nurse). One chimpanzee-usual gesture, Touch, was not produced by any infants or partners in this study and was dropped from further analyses. The rates in each group are shown in Fig. 5. The ANOVA result just failed to reach significance, *F*(5, 45) = 2.39, *p* = .053, η_p_^2^ = .210. Due to the high effect size (Bakeman, 2020), however, we report the post hoc contrasts. The rate of chimpanzee-usual gestures in the UK infants was significantly different only from the rate in the Gombe chimpanzee infants, *p* = .009. The rate in the UK infants did not differ significantly from the Nso and Aka human infants nor from the PRI/Zoo and Home-raised chimpanzee infants (all *p*s > .41).

**Fig. 6:**
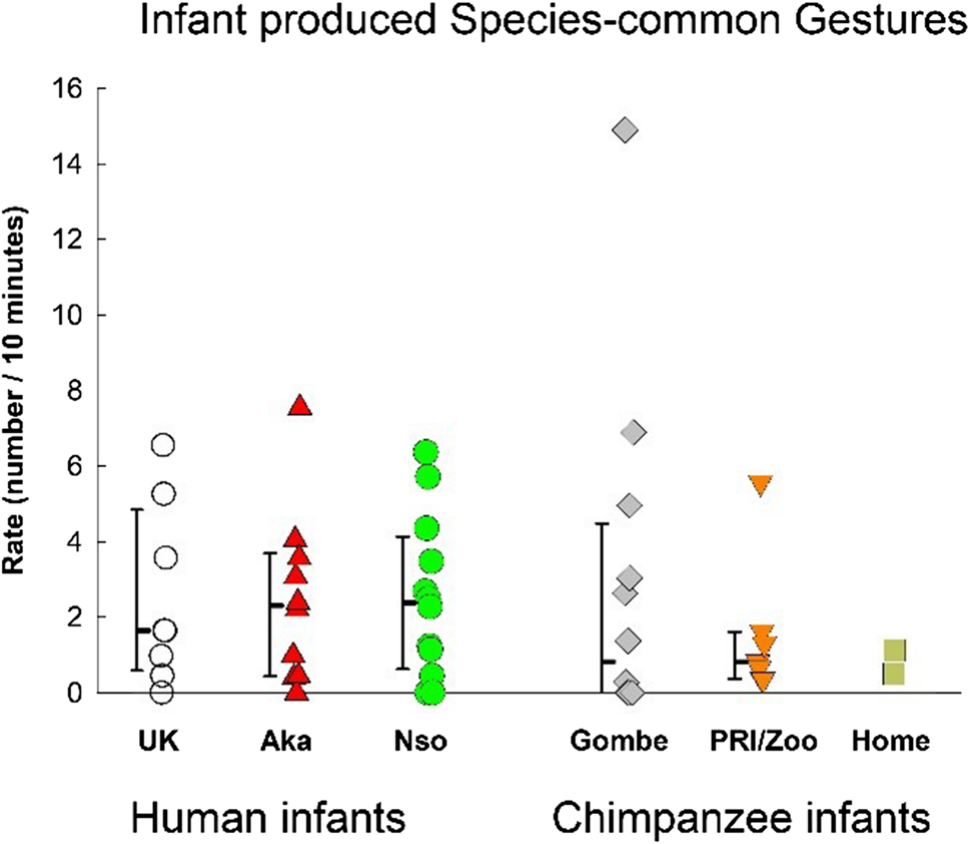
Infant produced species-common gestures (those gestures that are reported for both chimpanzee and human infants), across three human groups and three chimpanzee samples. Individual rates (number per 10 minutes of visible time) are shown with medians and 25–75% confidence intervals for each sample

We considered those infant-produced gestures that are reported in both the developmental and comparative literatures (arm raise, travel, request comfort, grunt, whine, request help, and other—which included hold mutual gaze) as “species-common” gestures. Rates in each group are displayed in Fig. 6. There were no significant differences across groups, *F*(5, 45) = 0.316, *p* = .901, η_p_^2^ = .034, or across species, *F*(1, 49) = .134, *p* = .716, η_p_^2^ = .003.

**Fig. 7:**
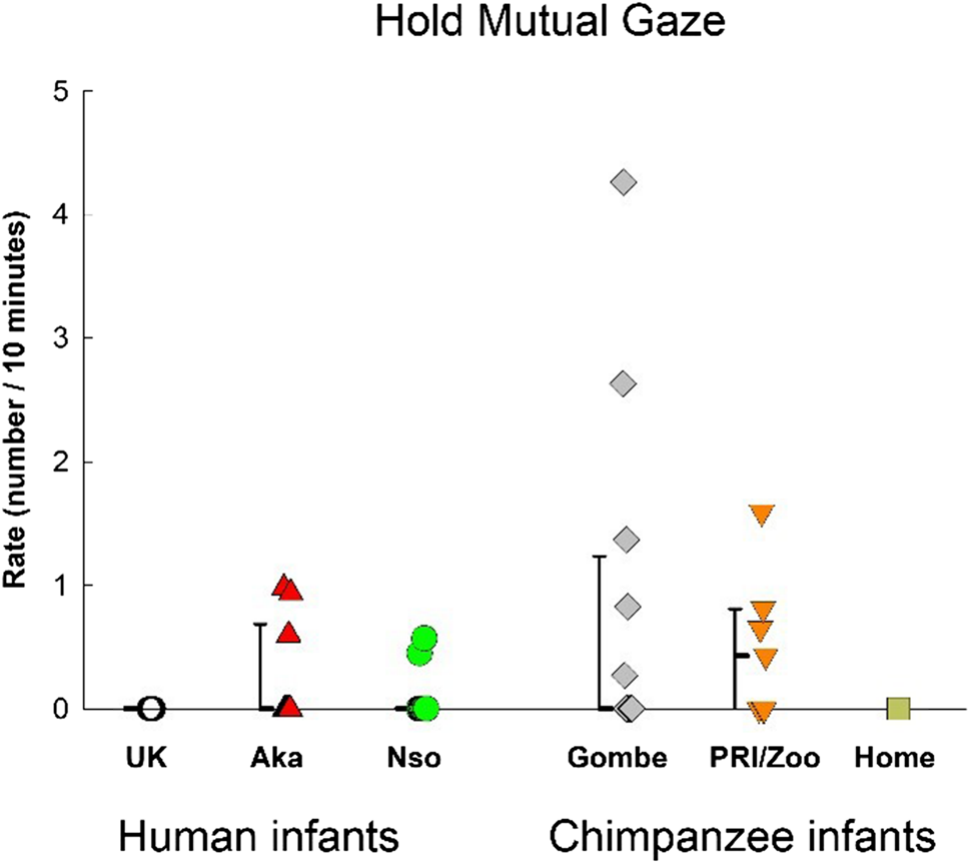
Hold mutual gaze for the three human infant samples (UK, Nso, Aka), and the three chimpanzee infant samples (Gombe, PRI/Zoo, Home-raised) in our study. Individual rates (number per 10 minutes of visible time) are shown with medians and 25–75% confidence intervals for each sample

Finally, we analyzed rates of the single behavior of Holding Mutual Gaze (Hold MG), a gesture that is thought to convey meaningful directedness. We found no difference among the groups (Fig. 7), *F*(5, 45) = 1.652, *p* = .166, η_p_^2^ = .155. But we found that the chimpanzee infants engaged in this gesture at significantly higher rates (.61 gestures/10min) than the human infants (0.12 gestures/10min), *F*(1, 49) = 5.678, *p* = .021, η_p_^2^ = .104. We found a significant positive correlation between the rate of Hold MG and the rates of infant-produced chimpanzee-usual gesture, *rho*(51) = .423, *p =* .002, and species-common gestures, *rho*(51) = .299, *p* = .033, but no significant correlation with infant produced human-usual gestures, *rho*(51) = −.247, *p* = .08.

**Fig. 8:**
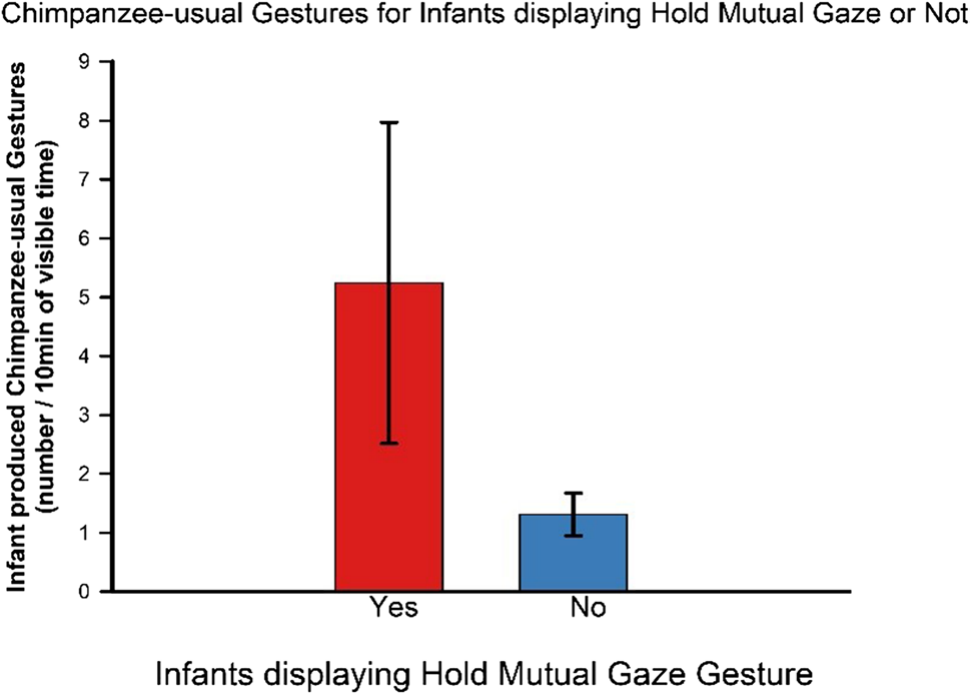
Chimpanzee-usual gestures were produced at significantly higher rates by infants who displayed the hold mutual gaze compared with those who did not display the Hold MG gesture (Mean & **SE**)

Given the relative high number of infants that did not produce any Hold MG gestures, we conducted a post hoc ANOVA using presence or absence of the hold-mutual-gaze gestures as a grouping variable. We found a significantly higher rate of species-common gestures in those infants who displayed Hold MG (*M* = 3.69, *SD* = 3.98, *n* = 14) than those did not display any Hold MG gestures (*M* = 1.88, *SD* = 2.01, *n* = 37), *F*(1, 49) = 4.63, *p* = .036, η_p_^2^ = .086, and significantly higher rates of chimpanzee-usual gestures in those infants who displayed Hold MG gestures than those who did not *F*(1, 49) = 5.261, *p* = .026, η_p_^2^ = .097 (Fig. 8).

### Gestural environment: Different types of infant-received gestures

**Fig. 9:**
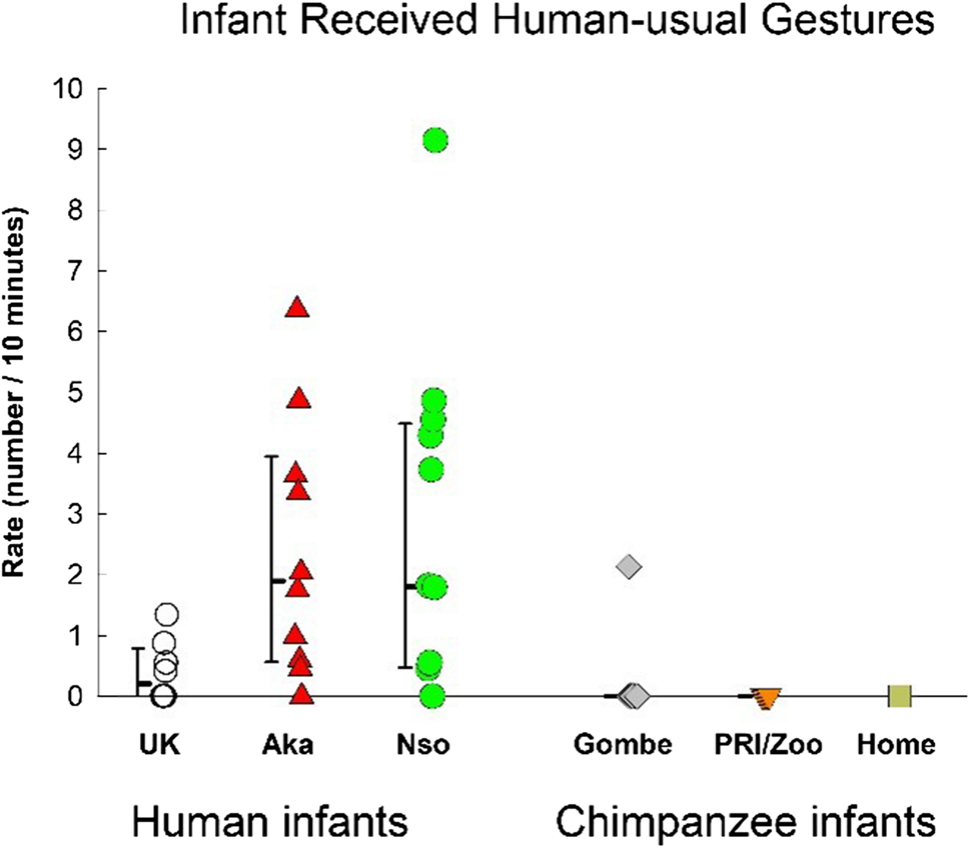
Infant received human-usual gestures for the three groups of human infants (UK, Nso, and Aka) and the three groups of chimpanzee infants (Gombe, PRI/Zoo, and Home-raised). Individual rates (number per 10 minutes of visible time) are shown with medians and 25–75% confidence intervals for each sample

Although there was no significant difference between species or among groups in the overall rate of infant-received gestures (see statistics reported in the section of Total Gestures & Fig. 3), there were group differences in the omnibus MANOVA containing the three types of infant-received gestures, *F*(15, 135) = 2.239, *p* = .008, η_p_^2^ = .199. For infant-received human-usual gestures (Fig. 9), there was a significant group difference, *F*(5, 45) = 4.883, *p* = .001, η_p_^2^ = .352. The UK group experienced significantly lower rates than the other human infant groups (Nso, *p* = .006; Aka, *p* = .016), but did not differ from the chimpanzee groups (all *p*s > .65).

**Fig. 10:**
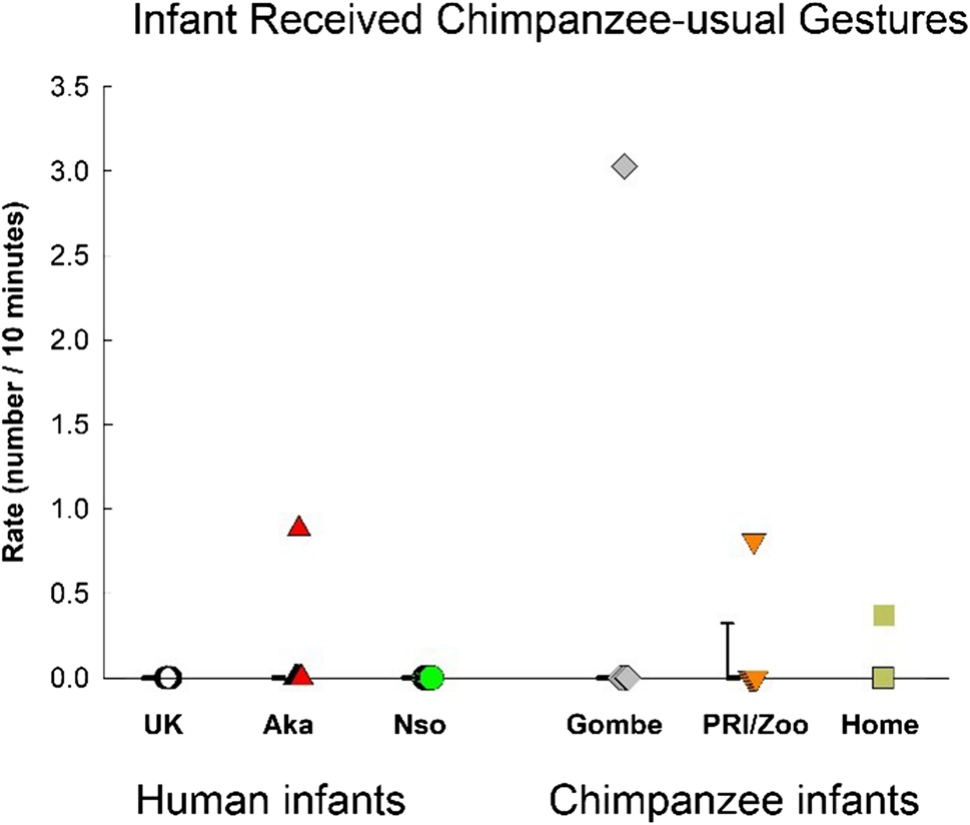
Infant-received chimpanzee-usual gestures for the three groups of human infants (UK, Nso, and Aka) and the three groups of chimpanzee infants (Gombe, PRI/Zoo, and Home-raised). Individual rates (number per 10 minutes of visible time) are shown with medians and 25–75% confidence intervals for each sample

The rates of infant-received chimpanzee-usual gestures is shown in Fig. 10. There was no significant difference among the groups, *F*(5, 45) = 0.489, *p* = .78, η_p_^2^ = .052, or between the species, *F*(1, 49) = 2.243, *p* = .14, η_p_^2^ = .044.

**Fig. 11:**
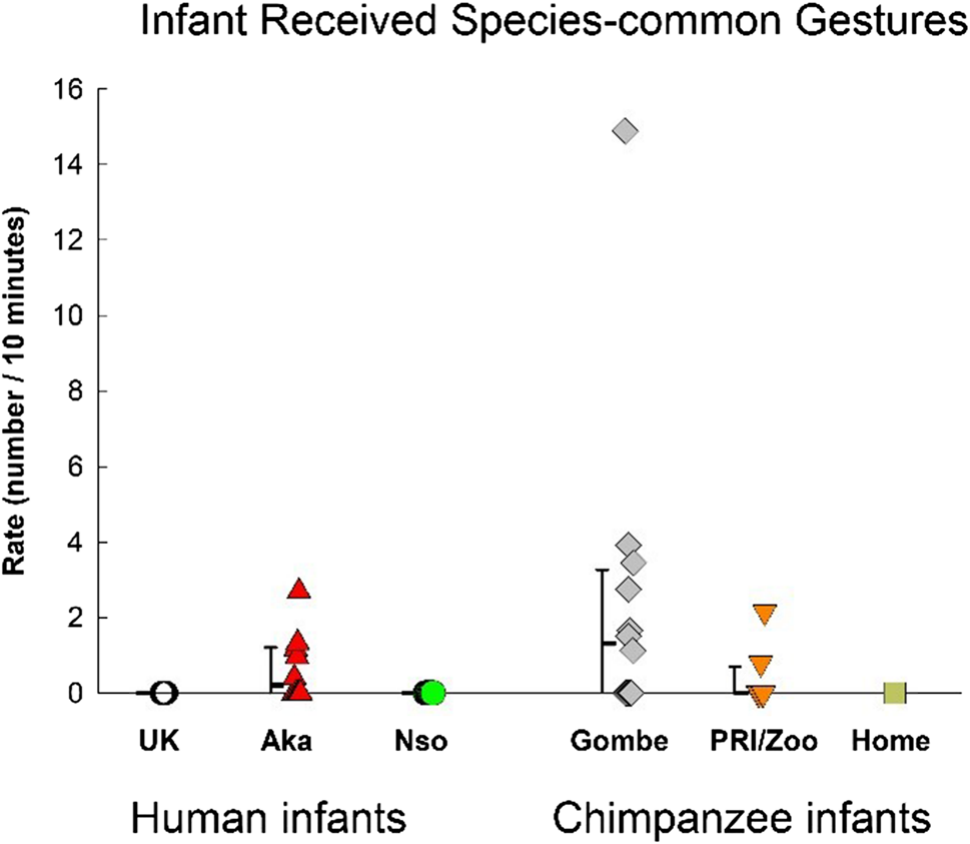
Infant-received species-common gestures for the three groups of human infants (UK, Nso, and Aka) and the three groups of chimpanzee infants (Gombe, PRI/Zoo, and Home-raised). Individual rates (number per 10 minutes of visible time) are shown with medians and 25–75% confidence intervals for each sample

The rates of infant-received species-common gestures are shown in Fig. 11. The difference among the groups just failed to reach significance, *F*(5, 45) = 2.109, *p* = .082, η_p_^2^ = .19. The UK group differed significantly only from the Gombe chimpanzees (*p* = .015), and not from the other chimpanzee infant groups, or from the other human infant groups.

We ran bivariate correlations to investigate the extent to which the gesture rates received by infants correlated with those produced by infants. A significant correlation between produced and received was found in the human-usual gesture types, *rho*(51) = .292, *p* = .038, but not in either the chimpanzee-usual gesture types, *rho*(51) = .129, *p* = .365, or the species-common gesture types, *rho*(51) = .009, *p* = .95.

SpeciesGiven that not all infants received all types of gestures, we additionally ran analyses focusing on the number of infants receiving each type of gesture, rather than comparing rates. We considered the local gesture environment by comparing the number of infants in each group that received each type of gestures. For these analyses, however, there were too many cells with low counts to run the analysis by group (we display the counts in Tables 2, 3, and 4). Therefore, we report the analyses only by species. We found a significant species difference in receiving human-usual gestures (Table 2), χ^2^(1) = 25.64, *p* < .001: more human infants received human-usual gestures than expected, and fewer chimpanzee infants received human-usual gestures than expected. Table 3 shows the number of infants that received chimpanzee-usual gestures by group. Since even in the analysis by species, the expected count for half the cells was under 5, we used a Fisher’s Exact Test, which was not significant, *p* = .15.

**Table 2:** The number of infants of each group (a) and species (b) that received human-usual gestures

Infant received Human-usual gestures								
(a) number of individuals		Group						Total
		UK	Nso	Aka	Gombe	PRI/Zoo	Home	
	YES	4	10	9	1	0	0	24
	NO	4	2	1	11	7	2	27
(b) Chi square association with Species								
				Yes	No	Total		
	Species	Human	Count	23	7	30		
			Expected Count	14.1	15.9	30.0		
		Chimpanzee	Count	1	20	21		
			Expected Count	9.9	11.1	21.0		
Total			Count	24	27	51

**Table 3:** The number of infants in each group that received chimpanzee-usual gestures

		group						Total
		UK	Nso	Aka	Gombe	PRI/Zoo	Home	
Number per group of Received Chimpanzee-usual gestures	YES	0	0	1	1	2	1	5
	NO	8	12	9	11	5	1	46

**Table 4:** The number of infants in each group (a) and each species (b) that received species-common gestures

Infant received Species-common gestures								
(a) number of individuals		Group						Total
		UK	Nso	Aka	Gombe	PRI/Zoo	Home	
	YES	0	0	5	7	2	0	14
	NO	8	12	5	5	5	2	37
(b) Chi square association with species								
				Yes	No	Total		
	Species	Human	Count	5	25	30		
			Expected Count	8.2	21.8	30.0		
		Chimpanzee	Count	9	12	21		
			Expected Count	5.8	15.2	21.0		
	Total		Count	14	37	51		
			Expected Count	14.0	37.0	51.0		

Finally, Table 4 shows the number of infants in each group that received species-common gestures. There was a significant effect of species on these gestures, χ^2^(1) = 4.255, *p* = .04. Fewer human infants received species-common gestures than expected and more chimpanzee infants received species-common gestures than expected (Table 4).

## Discussion

In this paper, we report infant-produced and infant-received gesturing as observed in samples of 1-year-old human and chimpanzee infants. The sample sizes were small and there were only three samples of human infants and three samples of chimpanzee infants, although there was diversity in socioecological settings within each species. Nonetheless, this study represents one of the only studies to make species comparisons in gestures while also documenting within-species variation in gestures. There were some findings that were expected, such as infants with human interactants produced higher rates of human-usual gestures than groups without human interactants, and infant groups with chimpanzee interactants produced significantly higher rates of chimpanzee-usual gestures than those without chimpanzee interactants. That said, we found fewer differences than expected. There were no differences among groups or across species in the overall rate of infant-produced gesturing or in the overall rate of infant-received gesturing. There were no significant differences across groups or species in the rates of gestures common to both species, whether infant produced or received. The most unexpected finding, however, was in the species difference in the behavior of Holding Mutual Gaze. Counter to theoretically driven expectations of the importance of mutual gaze for human infants raised with distal caregiving (see discussion in Akhtar & Gernsbacher, 2008), we found that the Holding Mutual Gaze gesture was more strongly associated with chimpanzees (who displayed significantly higher rates than human infants) and chimpanzee-usual gestures (infants who displayed the hold-mutual-gaze gesture produced significantly higher rates of chimpanzee-usual gestures compared with those infants who did not display the hold-mutual-gaze gesture). Although the communicative environments differed across groups in various ways, we indexed this with infant-received gestures. We found within-species differences in infant-received rates both of human-usual gesture types and of species-common gesture types. There was not, however, a tight association between infant-received and infant-produced gesturing. We found little correlation between the type of gestures received by infants and the type of gestures produced by infants, although we note that we only observed infants at a single age point and used only infant-received gestures as a broad index of the communicative environment.

Our premise in beginning this study was that previous communicative interactions were vital for the emergence of communicative gestures at 1 year of age, and that this would be true for chimpanzees as well as humans. One could presume that human infants would produce significantly more gestures than do chimpanzee infants because of the species differences in adult communicative competence, or that chimpanzee infants would produce more gestures that human infants because chimpanzee do not have any other means of communication (i.e., humans have language in addition to gestures, whereas chimpanzees have only gestures for communication). As infants, however, we found no support for either of these assumptions, since 1-year-old human and chimpanzee infants did not differ in their overall rates of produced gestures. One could also presume that human infants would receive significantly more gestures than do chimpanzee infants, based on the species differences in adult communicative competence. We found no support for this assumption, as 1-year-old human and chimpanzee infants did not differ in overall rates of received gestures.

The lack of species differences in infant-produced gestures could be related to differential rates of communicative development (e.g., 1-year-old human infants typically develop some spoken language while they are still communicating primarily with gestures, whereas chimpanzee infants typically communicate only nonverbally). Although human infants may be acquiring single words at 1 year of age, we find that in naturalistic observations their non-verbal communication rate is not different from that of chimpanzee infants. Although chimpanzee infants, given language training, may also express single words (e.g., Gardner & Gardner, 1998; Kellogg, 1968), none of our sample was language trained. We did not find an overall species difference in the rate of infant gesturing, the overall average rate was 5.9 gestures per 10 visible minutes at 1 year of age, but we do not know whether developmental trajectories of gesture rates may differ across species.

An important feature of our study was that we included three categories of gestures, those that are commonly reported for human infants, those that are commonly reported for chimpanzees, and those that are commonly reported for both species. We searched for within-species differences in these three types of gesture, which we expected to find, in part, because previously, Bard et al. (2021) found significant within species differences in the form of coordinated joint engagements, using these same naturalistic observations. Although the 1-year-olds in the Bard et al. (2021) study engaged in triadic co-ordinations for an average of 65% of the observation time, we found that the overall rate of gesturing in our samples of 1-year-olds was low. In fact, even with multiple gestures of each type, we found that not all infants produced (or received) all types of gesture. We were unable to document significant *within-species* differences for most gesture types but did document some species differences in types of gesture.

Since our study was only a “point-in-time,” we do not know how gesture rate trajectories may differ over time, nor, from this study, the processes by which gestures emerge (see Bard, Dunbar, et al., 2014b, for discussion of the range of processes by which gestures may develop). We do know that, in general, human infants’ verbal production increases, and chimpanzees fine tune their gesture repertoire from ~5 years to adulthood, using fewer but more successful gestures (Hobaiter & Byrne, 2011). Further discussion about linguistic relative to non-linguistic communication rates and how this may differ across species is beyond the scope of the present study.

### Infants’ communicative gesture environment

We expected to find tight associations between types of gestures that infants produce and types that they receive. We did not find a significant correlation overall between infant-produced and infant-received gesture rates and found a significant correlation in only one of the three gesture types. The rate of human-usual gestures produced by infants was significantly correlated with the rate of human-usual gestures received by infants. This finding is supported by previous studies, which show that, in humans, the frequency of human-usual gestures (especially pointing) produced by infants is positively correlated with the frequency of those received from their caregivers (Kishimoto, 2017; Liszkowski & Tomasello, 2011). The human-usual gestures are thought to be a way for infants to share attention and interest about objects with each other (Liszkowski et al., 2004). Although it has been suggested that the experience of shared attention and interest through human-usual gestures by caregivers may facilitate the development of shared intentionality (Tomasello, 2019), we note that (1) the UK infants experienced significant lower rates of infant-received human-usual gestures than the other human groups, and in fact, their rates of infant-received human-usual gestures did not differ from any of the chimpanzee infant groups, and (2) infant chimpanzees do not differ from infant humans in the amount of coordinated shared attention (Bard et al., 2021). We might argue instead that the presence of communicative gestures in human and chimpanzee infants, with their equivalent propensity for shared joint engagements (Bard et al., 2021) supports a conclusion that, at 1 year of age, infants of both species are flexibly responsive to the communicative needs of their local settings. Communicative needs may differ within species, across socioecological settings (e.g., for humans: Kwon et al., 2018; Vogt et al., 2015; Wang & Vallotton, 2016; and for chimpanzees: Boesch, 2012; de Waal, 1982; Fröhlich et al., 2020; Girard-Buttoz et al., 2022; Mitani, 2020; Wrangham et al., 2016), and may differ substantially later in life, across species, as we note that with fully developed language, humans, as a species, excel in communication compared with the chimpanzee species.

### Ostensive communication

One of the interesting findings of the study was that infants engage in the hold-mutual-gaze gesture already at 1 year of age, and that the chimpanzee infants had significantly higher rates of holding mutual gaze than the human infants. Although there have been several comparative studies of mutual gaze between 3-month-old infants and caregivers (which occurs in the daily life in humans: Keller, 2003; and in chimpanzees; Bard et al., 2005), this is probably the first study to compare its occurrence in older infants between species. The fact that the rate is higher in chimpanzees compared with humans is a surprising result. It has long been believed that such communication is rare in chimpanzees and may be associated with a lack of joint attention (Tomonaga et al., 2004), but perhaps this is associated with captive settings, more than the complex socioecologies of wild settings (e.g., collaborative hunting is found in some wild chimpanzee communities: Boesch, 2012). We know, for instance, that the frequency of 3-month mutual gaze is highly influenced by environmental factors for both chimpanzees (Bard et al., 2005) and humans (Keller, 2007), and that triadic connectedness (the competence underlying 1-year-old’s joint attention) is present at the same high levels in both human and chimpanzee infants (i.e., 65% of the time: Bard et al., 2021). We expected that hold mutual gaze would vary with the extent of distal caregiving—that is, would vary within species. This gesture, however, was significantly and positively related to the rate of chimpanzee-usual gestures, and not to the extent of human-usual gestures (see Bard et al., 2021, for further discussion).

The present result could be interpreted in several ways—for example, related to the reciprocity of mutual gaze and gaze alternation, to gaze patterns that differ across socioecological settings, or to the reliance on nonverbal communication in infants. Holding gaze may be antithetical to gaze alternation, so that if more holding gaze is observed then this means there will be less gaze alternation. It may be that the strong encouragement for objects as shared topics, such as in Western middle-class settings, reduces the occurrence of mutual gaze, instead focusing on gaze alternations. However, gaze alternations are characteristic of the joint attention toward objects for both humans and chimpanzees (e.g., Bard, Bakeman, et al., 2014a; Bates et al., 1975; Leavens et al., 2009), and mutual gaze can occur in 3-month-olds at similar rates for humans and chimpanzees (Bard et al., 2005), although there is significant within species variation for both species (see Bard et al., 2021, for discussion). To resolve this issue, we would need to assess the degree to which gaze alternations to objects is inversely related to the hold-mutual-gaze gesture in 1-year-olds.

Infants experiencing distal caregiving (i.e., the UK human infants and the home-raised chimpanzees) are encouraged to jointly focus on objects, whereas infants experiencing more proximal caregiving (i.e., the Nso and Aka human infants, and the Gombe and PRI/Zoo chimpanzees of the current study) are encouraged to jointly focus on social activities, as well (Bard et al., 2021). There are known cultural differences in mutual gaze that parallel cultural differences in distal versus proximal caregiving, and also parallel cultural differences in type of stimulation. In WEIRD cultures such as UK, distal parenting values lots of mutual gaze, and caregivers draw attention to the surrounding objects (so-called object stimulation), whereas in other cultural settings, such as subsistence farming or foraging communities of the Nso or Aka, respectively, caregivers stimulate infants with movement (sometimes called “gymnastics”: Takada, 2020) and visual attention to others (Keller, 2003, 2007; Lavelli et al., 2019). Although mutual gaze has been documented to be more frequent in distal than proximal settings around 3 months, we are not as aware of differences in frequency of mutual gaze at 1 year of age. Blake et al. (2003) found no increase in the coordination of gesture with eye contact in 9- to 14-month-old Japanese infants, “casting doubt upon the usefulness of eye contact as a criterion for communicative gesture . . . at least in naturalistic studies” (p. 15).

A final potential explanation for the species difference in hold mutual gaze may reside in differences in the manner of indicating (i.e., indicating the target of a communicative event). Although it may be that pointing emerges in response to the referential problem space (when individuals desire out-of-reach items but are prevented from obtaining them directly, needing a communicative partner to retrieve or deliver them: Leavens, 2021; Leavens et al., 2005), once in the repertoire, points are very effective in indicating specific referents. If points are not (yet) in the repertoire, human infants can use other ways to indicate, including verbal labels and eye gaze. It is plausible that human infants are more able to use the additional methods of verbalization and pointing in addition to Hold MG, where the chimpanzee infants, without verbalizations, are only able to indicate referents with gaze. This claim, however, is undermined by the lack of a significant correlation between the rate of human-usual gestures and hold mutual gaze, and instead we found that chimpanzee-usual gestures were significantly correlated with the hold-mutual-gaze gesture. Clearly more detailed studies of mutual gaze, gaze alternations, and type of gesture across various socioecological settings are needed to explain the reasons for this finding, which we found intriguing.

### Summary

Our study suggests that the overall rate of infant-produced gestures and the overall rate of infant-received gestures do not differ among groups of human infants from diverse socioecological settings or among chimpanzee infants from diverse socioecological settings, or in fact, between the species. However, when we delve into different types of gesture, we find differences in the rates of human-usual gestures and of chimpanzee-usual gestures. We also found that there were differences in the rate of infant-received human-usual gestures. A surprising finding was that the chimpanzee infants engage in the hold-mutual-gaze gesture at a significantly higher rate than the human infants, which significantly correlated to the rate of chimpanzee-usual gestures, not human-usual gestures as might be expected from the importance of gaze that is suggested in the human developmental literature. Our study highlights the value of including a diverse range of gesture types in studies of early communication, and of comparing diverse groups of both species to aid in identifying those aspects of gestural communication which may differ within and between species.
